# The possible association between epidemics of hand-foot-and-mouth disease and responsiveness to immunoglobulin therapy in Kawasaki disease

**DOI:** 10.3389/fped.2022.968857

**Published:** 2022-09-06

**Authors:** Daisuke Shimizu, Takayuki Hoshina, Masaru Kawamura, Yoshie Tomita, Yasufumi Hidaka, Masumi Kojiro, Jun Muneuchi, Junji Kamizono, Kenichiro Yamaguchi, Yoshihisa Fujino, Koichi Kusuhara

**Affiliations:** ^1^Department of Pediatrics, University of Occupational and Environmental Health, Kitakyushu, Japan; ^2^Department of Pediatrics, National Hospital Organization Kokura Medical Center, Kitakyushu, Japan; ^3^Department of Pediatrics, Kitakyushu Municipal Yahata Hospital, Kitakyushu, Japan; ^4^Department of Pediatrics, Kitakyushu Municipal Medical Center, Kitakyushu, Japan; ^5^Department of Pediatrics, Kitakyushu General Hospital, Kitakyushu, Japan; ^6^Department of Pediatrics, Japan Community Health Care Organization Kyushu Hospital, Kitakyushu, Japan; ^7^Department of Environmental Epidemiology, School of Medicine, Institute of Industrial Ecological Science, University of Occupational and Environmental Health, Kitakyushu, Japan

**Keywords:** Kawasaki disease (KD), hand-foot-and-mouth disease (HFMD), enterovirus (EV), responsiveness to immunoglobulin therapy, negative correlation

## Abstract

**Background:**

Enterovirus causing hand-foot-mouth disease (HFMD) has been reported to be associated with the development of Kawasaki disease (KD), whereas the involvement of enterovirus in the clinical course of KD is uncertain. The aim of this study is to investigate the association between the clinical course of KD and HFMD epidemics.

**Methods:**

This study included 108 patients who developed KD during HFMD epidemic seasons (July and August) from 2010 to 2014 and who were initially treated with high-dose intravenous immunoglobulin (IVIG). A mean of ≥5.0 HFMD patients reported weekly from each sentinel medical facility was considered to represent a large HFMD epidemic. We compared the clinical characteristics of KD patients in summers of years with and without large HFMD epidemics.

**Results:**

Large HFMD epidemics occurred in 2011 and 2013. The number of KD patients in summer was the highest in 2011. The proportion of patients with resistance to the IVIG therapy in summers of years with the large epidemics (14%) was significantly lower than that in summers of other years (31%, *P* = 0.030), whereas the proportion of patients with coronary artery abnormalities did not differ to a statistically significant extent. The development of KD during large HFMD epidemics was significantly associated with a lower risk of resistance to the IVIG therapy (incidence rate ratio 0.92, *P* = 0.049).

**Conclusion:**

Patients developing KD during large HFMD epidemic may have good responsiveness to IVIG. It is important to identify microbes from KD patients to predict responsiveness to IVIG therapy.

## Introduction

Kawasaki disease (KD) is a self-limiting systemic vasculitis of unknown etiology that occurs in infants and children (1). Currently, KD is considered to be a disease that results from the exposure of genetically susceptible individuals to infectious or environmental triggers (2). KD epidemics have been reported and the disease shows seasonality (3–6). Thus, it has been hypothesized that various microbes might be associated with the development of KD. Previous studies have suggested that genetic factors might also be associated with susceptibility to the development of coronary artery abnormalities (CAAs) or resistance to high dose intravenous immunoglobulin (IVIG) therapy (7, 8). On the other hand, the association of microbes with the development of complications or responsiveness to therapy remains uncertain.

We previously reported, based on a study (all the patients were Japanese) conducted in a Japanese metropolitan city, that there was seasonality in the responsiveness to the initial IVIG therapy in KD patients, and that the proportion of KD patients with resistance to the treatment peaked in the warm period, especially summer (9). This result suggested that some environmental triggers, including microbes, might be associated with responsiveness to IVIG therapy in KD patients. In that study, we found that the number of KD patients peaked in winter in most years, while it only peaked in summer in 2011. In addition, it seemed that the proportion of KD patients with resistance to the initial IVIG therapy in the summer of the year was lower in comparison to all other years. Meanwhile, we noted that a large epidemic of hand-foot-mouth disease (HFMD) occurred in 2011 (10). Enterovirus, a causative pathogen of HFMD, is a prevalent virus in summer and has been reported to be associated with the development of KD (11–13). Based on the results of our previous study and the epidemiological findings, we hypothesized that HFMD might be related to the responsiveness to IVIG therapy in KD patients.

To investigate the association between the clinical course of KD and HFMD epidemics, we compared the clinical characteristics of KD patients in the summers of the years with and without large HFMD epidemics.

## Materials and methods

### The investigation of HFMD epidemics and the definition of “large HFMD epidemic”

To assess HFMD epidemics during the study period, we investigated the total number of cases with HFMD reported from 24 sentinel medical facilities in Kitakyushu City with reference to the infectious disease surveillance data of the city (10). HFMD had been prevalent every year from the end of June to the end of August. Thus, we defined the HFMD epidemic period as July to August. Furthermore, if the mean number of HFMD patients reported weekly from each facility was ≥5.0, we determined that a large epidemic had occurred in the week. A year in which the mean weekly number of reported HFMD cases exceeded 5.0 even once was defined as “year with a large epidemic.”

### Study population

From 2010 to 2014, 744 KD patients were admitted to the pediatrics departments of 6 hospitals in Kitakyushu City. The eligible patients were also enrolled in our previous study to investigate the seasonality of the clinical course of KD (9). Among these patients, 108 who developed KD in July or August and who were initially treated with high-dose IVIG were enrolled in the present retrospective study. We compared the demographic and clinical characteristics between children who developed KD during the HFMD epidemic periods in years with large epidemics and those who developed KD in other years. The clinical information on each patient was collected using a standardized case report form. The Diagnostic Guidelines for Kawasaki Disease (fifth version) were applied as the diagnostic criteria for KD (14). High-dose IVIG (2 g/kg/dose) and oral aspirin (30 mg/kg/day) were administered as the initial treatments for KD. IVIG was administered in 12–24 h. None of the patients with response to the initial IVIG therapy received corticosteroid or other medication such as infliximab and cyclosporin A. Our study was approved by the Institutional Review Board of the University of Occupational and Environmental Health, Japan (ID: H28-063).

### Evaluations

The analyzed data included the age (in months), the day of illness at the initial treatment, the laboratory data, the score for predicting resistance to the initial IVIG therapy (Kobayashi score) (15), and the proportions of patients with resistance to initial IVIG therapy and those with coronary artery abnormalities (CAAs) assessed by echocardiography. The laboratory data included the peripheral white blood cell, neutrophil, and platelet counts, and the serum aspartate aminotransferase, sodium and C-reactive protein (CRP) levels which were measured before the initial treatment. Patients whose body temperature was ≥37.5°C for ≥24 h after the initial IVIG therapy or who showed recurrent KD symptoms after initial defervescence were considered to be resistant to the initial IVIG therapy (14). CAA was defined by an internal lumen diameter of ≥4 mm or local dilation (≤ 4 mm internal diameter in children of <5 years of age and <1.5 fold that of an adjacent segment in children of ≥5 years of age) (16).

### Statistical analyses

The Mann-Whitney *U*-test was used to compare quantitative values and the chi-squared test and Fisher's exact test were used for the qualitative analyses. Incidence rate ratios (IRR) of the mean number of HFMD patients reported weekly from each facility for the resistance to the initial IVIG therapy were estimated by Poisson regression in which the number of the development of KD was used as off-set. We also used a scatter plot to investigate the relationship between the responsiveness to IVIG treatment and the number of HFMD patients. The Stata software program (version 15, Stata Corporation, College Station, TX**)** was used to perform the statistical analyses. *P*-values of <0.05 were considered to indicate statistical significance.

## Results

The demographic and clinical characteristics of the enrolled patients are shown in Table 1. None of the patients had received IVIG therapy before the diagnosis of KD. The median age, male-to-female ratio and the median days of illness at the initial IVIG therapy of the enrolled patients were similar to those a recent Japanese nationwide survey (17). In the present study, the proportion of KD patients with resistance to the initial IVIG therapy (23.1%) was higher and the proportion of those with CAAs (3.7%) was lower in comparison to the results of the nationwide survey (19.7 and 9.0%, respectively). In HFMD non-epidemic periods excluding every July and August during the investigation period of our previous study (9), of the 607 KD patients, 110 (18.1%) were refractory to the initial IVIG therapy and 22 (3.6%) developed CAAs (data not shown).

**Table 1:** The demographic and clinical characteristics of KD patients with the initial IVIG therapy.

**Characteristics**	**Eligible patients, *n* = 108**
Age, months, median (range)	23.5 (2–87)
Gender, *n*, %male	56 (51.9)
Days of illness at the initial IVIG therapy, median (range)	5 (2–10)
Patients with resistance to the initial IVIG therapy, *n* (%)	25 (23.1)
Proportion of the IVIG resistance rate in patients with serum C-reactive protein ≥10 mg/L^*a^, %	24.2 (8/33)
Proportion of the IVIG resistance rate in patients with serum C-reactive protein <10 mg/L^*a^, %	22.7 (17/75)
Patients with CAAs, *n* (%)	4 (3.7)
Fatal case, *n* (%)	0 (0)

*a With reference to Kobayashi score (15).

KD, Kawasaki disease; IVIG, intravenous immunoglobulin; CAA, coronary artery abnormality.

**Figure 1:**
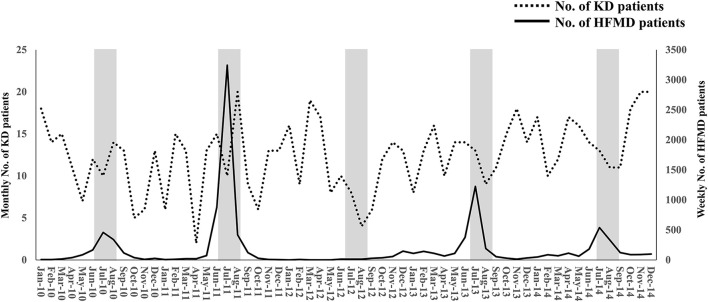
The monthly number of KD patients and the weekly number of HFMD patients during the study period. The weekly number of HFMD patients was reported from 24 sentinel medical facilities in Kitakyushu City. The left and right Y-axes indicate the monthly number of KD patients and the weekly number of HFMD patients, respectively. The gray zones indicate July and August. KD, Kawasaki disease; HFMD, hand-foot-mouth disease.

**Figure 2:**
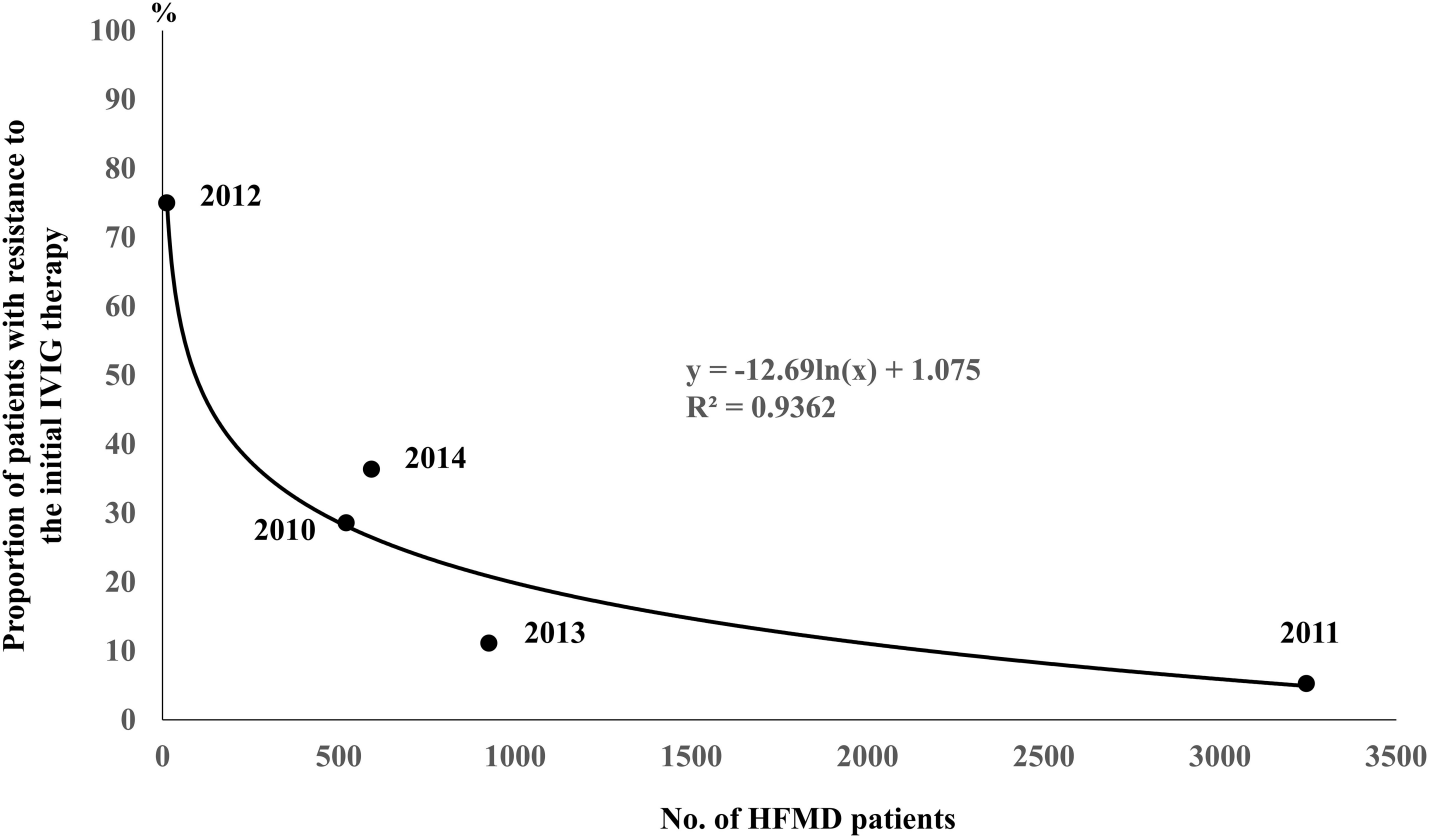
Correlation between the proportion of patients resistant to the initial IVIG therapy in those who developed KD in August and the total weekly number of HFMD patients reported from the sentinel medical facilities in Kitakyushu City in July. The ratios of the number of KD patients with resistance to the initial IVIG therapy to total number of KD patients in August of each year were 4/14 in 2010, 1/19 in 2011, 3/4 in 2012, 1/9 in 2013 and 4/11 in 2014. The numbers in the brackets indicate the numbers of total KD patients (left) and those with resistance to the initial IVIG therapy (right).

In HFMD epidemic periods, the highest number of KD patients was in 2011, when the number of HFMD patients was the highest, and the lowest number of KD patients was in 2012, when the number of HFMD patients was the lowest (Figure 1). During the study period, large HFMD epidemics occurred in 2011 and 2013 (Figure 1). The main serotypes of enterovirus isolated from HFMD patients were enterovirus 71 in 2010, coxsackievirus A16 in 2011, coxsackievirus A2, A5 and B4 in 2012, coxsackievirus A6 and enterovirus 71 in 2013, and coxsackievirus A6 and enterovirus 71 in 2014 (18). No endemic of infectious diseases other than HFMD was observed in the summer from 2010 to 2014 (10). We compared the demographic and clinical characteristics between patients who developed KD in July and August of 2011 or 2013 (summers of years with large epidemic, *n* = 50) and those who developed KD in July and August of 2010, 2012 or 2014 (summers of other years, *n* = 58). The serum CRP levels were higher in summers of years with large epidemic than of other years (*P* = 0.015). With the exception of the CRP level, there were no significant differences in the demographic and laboratory data or in the median days of illness at the initial IVIG therapy between the two groups (Table 2). The proportion of patients with resistance to the initial IVIG therapy in summers of years with large epidemic (14%) was significantly lower than that in summers of other years (31%, *P* = 0.030). The IVIG resistance rate in the summer of HFMD epidemic years was also lower than that for the entire investigation period (19%, data not shown). The proportion of patients with CAAs did not differ between the two groups to a statistically significant extent. No patients died during the study period. The association between the number of patients with resistance to the initial IVIG therapy in each July or August and the mean number of HFMD patients reported weekly from each sentinel medical facility in each previous month was examined using a Poisson regression analysis. There was a negative association between the two variables (IRR 0.92, 95% confidence interval 0.86–0.99, *P* = 0.049, Table 3). As it is possible that there was a time lag between the onset of the diseases directly induced by pathogens, such as HFMD, and diseases indirectly induced by pathogens, such as KD, we also compared the correlation between the proportion of patients who were resistant to the initial IVIG therapy those who developed KD in August and the total weekly number of HFMD patients reported from the sentinel medical facilities in Kitakyushu City in July. A marked negative correlation was indicated in the relationship between the two variables (R^2^ = 0.9362, Figure 2).

**Table 2:** The comparison of the demographic and clinical characteristics between patients who developed KD during summers in large HFMD epidemic years and those in other years.

**Characteristics**	Large epidemic years^*a^, *n* = 50	other years^*b^, *n* = 58	***P*-value**
Age, months, median (range)	23.5 (3–87)	23 (2–70)	0.258
Gender, *n*, %male	29 (58.0)	27 (46.6)	0.235
Days of illness at the initial IVIG therapy, median (range)	5 (3–10)	5 (2–8)	0.437
White blood cell counts, 10^9^/L, median (range)	13.3 (6.3–23.2)	12.3 (7.7–33.8)	0.687
Neutrophil, %, median (range)	67.4 (20.5–94.2)	68.6 (23.7–88.0)	0.526
Platelet counts, ×10^9^/L, median (range)	329 (129–824)	298 (154–660)	0.091
Serum aspartate aminotransferase level, IU/L, median (range)	35.5 (21.0–4489)	43.0 (16.0–750)	0.968
Serum sodium level, mEq/L, median (range)	135 (128–144)	135 (124–143)	0.342
Serum C-reactive protein level, mg/L, median (range)	58 (17–279)	89 (8–269)	0.015
Kobayashi score, median (range)	3 (0–10)	3 (0–10)	0.188
Patients with resistance to the initial IVIG therapy, *n* (%)	7 (14.0)	18 (31.0)	0.030
Patients with CAAs, *n* (%)	2 (4.0)	2 (3.4)	0.880
Fatal case, *n* (%)	0 (0)	0 (0)	N.D.

We investigated data on KD patients that developed in July and August from 2010 to 2014.

A year in which the mean weekly number of HFMD cases reported from each sentinel medical facility (n = 24) exceeded 5.0 even once was defined as “large epidemic year.” The large HFMD epidemic years were 2011 and 2013.

*a Patients who developed KD in 2011 or 2013 were classified into this category.

*b Patients who developed KD in 2010, 2012 or 2014 were classified into this category.

IVIG, intravenous immunoglobulin; CAA, coronary artery abnormality; N.D., no data.

**Table 3:** Association of HFMD epidemic for the number of KD patients with resistance to the initial IVIG therapy.

	**IRR**	**95% CI**	***P*-value**
The mean weekly number of HFMD cases from each sentinel medical facility	0.92	0.86–0.99	0.049

Incidence rate ratios (IRR) of the mean weekly number of HFMD cases from each sentinel medical facility (n = 24) for the resistance to the initial IVIG therapy were estimated by Poisson regression in which the number of the development of KD was used as off-set.

IRR, incident rate ratio; CI, confidence interval; IVIG, intravenous immunoglobulin; HFMD, hand-foot-mouth disease.

We additionally investigated the demographic and clinical characteristics between patients who developed KD during epidemic and non-epidemic periods in the years with large HFMD epidemics (2011 and 2013) by extracting data from our previous study (9). In contrast to the results of our previous study indicating that the proportion of KD patients with resistance to the initial IVIG therapy was peaked in the warm period, especially summer (9), the IVIG resistance rate was lower in KD patients developing during HFMD epidemic period (July and August) rather than during non-epidemic period, although not to a significant extent (Table 4).

**Table 4:** The comparisons of the demographic and clinical characteristics between patients who developed KD during epidemic and non-epidemic periods in the years of large HFMD epidemics^*a^.

**Characteristics**	Epidemic period^*b^ *n* = 50	Non-epidemic period^*c^ ***n* = 229**	***P*-value**
Age, months, median (range)	23.5 (3–87)	24.0 (1–143)	0.80
Gender, *n*, %male	29 (58.0)	140 (61.1)	0.68
Days of illness at the initial IVIG therapy, median (range)	5 (3–10)	5 (1–10)	0.37
White blood cell counts, 10^9^/L, median (range)	13.3 (6.3–23.2)	13.5 (1.1–33.0)	0.59
Neutrophil, %, median (range)	67.4 (20.5–94.2)	65.3 (5–92)	0.71
Platelet counts, ×10^9^/L, median (range)	329 (129–824)	299 (74–1,299)	0.20
Serum aspartate aminotransferase level, IU/L, median (range)	35.5 (21–4,489)	37.0 (4.1–874)	0.63
Serum sodium level, mEq/L, median (range)	135 (128–144)	135 (125–143)	0.51
Serum C-reactive protein level, mg/L, median (range)	58 (17–279)	68 (0.7–207)	0.27
Kobayashi score, median (range)	3 (0–10)	3 (0–10)	0.15
Patients with resistance to the initial IVIG therapy, *n* (%)	7 (14)	43 (18.7)	0.42
Patients with CAAs, *n* (%)	2 (4.0)	10 (3.6)	0.79
Fatal case, *n* (%)	0 (0)	0 (0)	N.D.

*a The years with large HFMD epidemics indicate 2011 and 2013.

*b Epidemic period indicates July and August.

*c Non-epidemic period indicated from January to June and from September to December.

HFMD, hand-foot-mouth disease; KD, Kawasaki disease; IVIG, intravenous immunoglobulin; CAA, coronary artery abnormality; N.D., no data.

## Discussion

Enterovirus, a causative pathogen of HFMD, has been reported to be associated with the development of KD. Three previous studies from Taiwan indicated that the number of KD patients markedly increased in the summers of years in which enterovirus 71 epidemics occurred (11), that a history of previous enterovirus infection in children of 3–5 years of age was associated with a higher risk developing KD (13), and that the detection rate of enterovirus in the upper respiratory tract of KD patients was significantly higher in comparison to age-matched healthy children (12). On the other hand, to the best of our knowledge, no reports have investigated the association between the clinical course of KD and HFMD epidemics. In the present study, a negative association was shown between the development of KD during HFMD epidemics and the risk of resistance to IVIG therapy, suggesting that patients developing KD during large HFMD epidemic might show good responsiveness to IVIG.

The administration of IVIG has been considered to be effective for the prevention of enterovirus infection and the treatment of severe enterovirus infection (19–21). In patients with agammaglobulinemia who show increased susceptibility to severe enterovirus infection, maintaining high titers of serum antibodies to enteroviruses by IVIG replacement was important for preventing infection (22). IVIG therapy was also effective for enterovirus-associated regional enteritis in a patient with X-linked agammaglobulinemia (23), suggesting that the neutralization of the virus by the administration of IVIG led to the improvement of the symptoms. Furthermore, IVIG therapy without concomitant corticosteroid therapy, was useful for the treatment of severe enterovirus 71 infection, including encephalitis and pneumonia (21). This finding indicated that immunoglobulin might suppress the hypercytokinemia caused by enterovirus infection. Given these previous reports, the results of the present study showing a higher proportion of KD patients with susceptibility to IVIG therapy in the summers of years with large HFMD epidemics indirectly indicate that immunoglobulin may be more effective for patients developing KD during large HFMD epidemic. The outbreak of a specific infectious disease could change the pattern of the seasonal variation of KD (12, 24, 25). In addition, the difference in responsiveness to the IVIG therapy among microbes may change the seasonal pattern of the clinical course of KD. In order to predict responsiveness to IVIG therapy, it will be important to try to identify various microbes from KD patients. Although the difference of the lot number of immunoglobulin might affect the efficacy of IVIG therapy for KD, in patients diagnosed with KD from April to June and from September to November 2011, the IVIG resistance rates were 22.2 and 17.9%, respectively, and higher than in those from July to August, indicating that the lot number of immunoglobulin is not considered to affect its efficacy. In addition, the difference of the brand of immunoglobulin may also affect the efficacy of IVIG therapy for KD. Although not all 6 hospitals use the same brand of immunoglobulin, the IVIG resistance rate was not significantly different among them.

In the present study, the serum CRP levels in patients who developed KD during the summers of years with large HFMD epidemic was significantly lower in comparison to the summers of other years. In addition, peripheral platelet counts in the former KD patients were higher than those in the latter patients although not to a statistically significant extent. Higher levels of serum CRP and lower platelet counts were risk factors for predicting resistance to IVIG therapy in KD patients (15, 26, 27). From the aspect of laboratory findings, it was also indicated that patients who developed KD during the summers of years with large HFMD epidemics showed good responsiveness to IVIG.

Compared with the nationwide survey (17), the proportion of KD patients with resistance to the initial IVIG therapy was higher in the summers of years without large HFMD epidemics. In our previous report (9), the proportion of KD patients with resistance to the initial IVIG therapy was peaked in the warm period, especially summer. The course of KD that is refractory to IVIG therapy is often severe. Based on the results of this study, as one hypothesis, we considered that the severity of KD might be affected by the variation in the ambient temperature. Increased temperatures provide optimal conditions for pathogen survival, virulence and replication as well as increased opportunities for human-pathogen interaction (28, 29). In addition, the elevation of ambient temperature favors transition of pathogens within the nasopharynx from the carrier state to invasive state (30). The increased loads of specific pathogens and the promotion of the invasion due to an increased ambient temperature may be also associated with the severity of KD. Given that the baseline IVIG resistance rate in the summer was high in the investigation period, even HFMD epidemic was associated with low resistant rate, it would be expected that the resistance rate in the summer was not substantially lower than that in non-summer months in HFMD endemic years.

The incidence of CAAs in this study (3.7%) was lower than that in the nationwide survey (9.0%) (17). As one of this reason, early additional therapies might be appropriately performed for the eligible KD patients with resistance to the initial IVIG therapy. Alternatively, it was speculated that there may be the regional difference in the incidence of CAAs, because environmental factors and epidemics of infectious diseases that are considered to be associated with the formation of CAAs may be different by regions. The similar study is needed to be performed in other regions in Japan.

The present study was associated with some limitations. First, the sample size was relatively small because the investigation period was not sufficient. This could have affected the accuracy of the statistical analysis. However, as our study was a post-enumeration survey in a region, we could not increase the subjects premeditatedly. Second, we could not confirm whether the eligible patients carried enterovirus at the onset of KD because this is an epidemiological study. Accordingly, the serotypes of enteroviruses were not investigated in these patients, and the specific serotypes associated with the development of KD remain uncertain. In addition, we could not collect data on history of recent enterovirus infection in the eligible KD patients of this retrospective study. A long-term prospective study is needed to clarify the association between enterovirus infection and responsiveness to IVIG therapy in KD patients.

In conclusion, the present study indicated that the proportion of KD patients with resistance to the initial IVIG therapy was significantly lower in the summers of years with large HFMD epidemic than in the summers of other years. Furthermore, a negative association was identified between the development of KD during HFMD epidemics and the risk of resistance to IVIG therapy. This result suggested that patients developing KD during large HFMD epidemic might show good responsiveness to IVIG. Epidemiological studies may be useful not only for linking two unrelated diseases but also for predicting responsiveness to treatment. A further prospective study is desired to investigate the relationship between KD and environmental triggers such as HFMD.

## Data availability statement

The raw data supporting the conclusions of this article will be made available by the authors, without undue reservation.

## Ethics statement

The studies involving human participants were reviewed and approved by the Institutional Review Board of the University of Occupational and Environmental Health, Japan. Written informed consent from the participants' legal guardian/next of kin was not required to participate in this study in accordance with the national legislation and the institutional requirements.

## Author contributions

DS and TH conceptualized the study, carried out the initial analysis of data for work, drafted the initial manuscript, and approved the final manuscript as submitted. MKa, YT, YH, MKo, JM, JK, KY, and YF carried out the initial analyses, reviewed and revised the manuscript, and approved the final manuscript as submitted. KK designed the study, reviewed and revised the manuscript, and approved the final manuscript as submitted. All authors contributed to the article and approved the submitted version.

## Conflict of interest

The authors declare that the research was conducted in the absence of any commercial or financial relationships that could be construed as a potential conflict of interest.

## Publisher's note

All claims expressed in this article are solely those of the authors and do not necessarily represent those of their affiliated organizations, or those of the publisher, the editors and the reviewers. Any product that may be evaluated in this article, or claim that may be made by its manufacturer, is not guaranteed or endorsed by the publisher.

## References

[1] Burns JC Glodé MP. Kawasaki syndrome. Lancet. (2004) 364:533–44. 10.1016/S0140-6736(04)16814-1 15302199

[2] Hara T Nakashima Y Sakai Y Nishio H Motomura Y Yamasaki S. Kawasaki disease: a matter of innate immunity. Clin Exp Immunol. (2016) 186:134–43. 10.1111/cei.12832 27342882 PMC5054572

[3] Galeotti C Bayry J Kone-Paut I Kaveri SV. Kawasaki disease: aetiopathogenesis and therapeutic utility of intravenous immunoglobulin. Autoimmun Rev. (2010) 9:441–8. 10.1016/j.autrev.2009.12.004 20004744 PMC7185630

[4] Burns JC Herzog L Fabri O Tremoulet AH Rodó X Uehara R. Seasonality of Kawasaki disease: a global perspective. PLoS ONE. (2013) 8:e74529. 10.1371/journal.pone.0074529 24058585 PMC3776809

[5] Makino N Nakamura Y Yashiro M Kosami K Matsubara Y Ae R. Nationwide epidemiologic survey of Kawasaki disease in Japan, 2015-2016. Pediatr Int. (2019) 61:397–403. 10.1111/ped.13809 30786118

[6] Rodó X Curcoll R Robinson M Ballester J Burns JC Cayan DR. Tropospheric winds from northeastern China carry the etiologic agent of Kawasaki disease from its source to Japan. Proc Natl Acad Sci USA. (2014) 111:7952–7. 10.1073/pnas.1400380111 24843117 PMC4050536

[7] Onouchi Y Suzuki Y Suzuki H Terai M Yasukawa K Hamada H. *ITPKC* and *CASP3* polymorphisms and risks for IVIG unresponsiveness and coronary artery lesion formation in Kawasaki disease. Pharmacogenomics J. (2013) 13:52–9. 10.1038/tpj.2011.45 21987091

[8] Shimizu C Eleftherohorinou H Wright VJ Kim J Alphonse MP Perry JC. Genetic variation in the SLC8A1 calcium signaling pathway is associated with susceptibility to Kawasaki Disease and coronary artery abnormalities. Circ Cardiovasc Genet. (2016) 9:559–68. 10.1161/CIRCGENETICS.116.001533 27879314

[9] Shimizu D Hoshina T Kawamura M Tomita Y Hidaka Y Kojiro M. Seasonality in clinical courses of Kawasaki disease. Arch Dis Child. (2019) 104:694–6. 10.1136/archdischild-2018-315267 30413486

[10] The Infectious Disease Occurrence Trend (fixed point report) (homepage on the Internet). City of Kitakyushu. Available online at: https://kitakyushu.j-server.com/LUCKKYUSHU/ns/tl.cgi/https://www.city.kitakyushu.lg.jp/ho-huku/18300149.html?SLANG=ja&TLANG=en&XMODE=0&XPARAM=pageIdSearchtxt,&XCHARSET=UTF-8&XPORG=,&XJSID=0 (accessed May 12, 2022).

[11] Chang LY Chang IS Lu CY Chiang BL Lee CY Chen PJ. Epidemiologic features of Kawasaki disease in Taiwan, 1996-2002. Pediatrics. (2004) 114:e678–682. 10.1542/peds.2004-0726 15574600

[12] Chang LY Lu CY Shao PL Lee PI Lin MT Fan TY. Viral infections associated with Kawasaki disease. J Formos Med Assoc. (2014) 113:148–54. 10.1016/j.jfma.2013.12.008 24495555 PMC7125523

[13] Weng KP Cheng-Chung Wei J Huang SH Chien KJ Lin CC. Enterovirus infection and subsequent risk of Kawasaki Disease: a population-based cohort study. Pediatr Infect Dis J. (2018) 37:310–5. 10.1097/INF.0000000000001748 28834956

[14] Ayusawa M Sonobe T Uemura S Ogawa S Nakamura Y Kiyosawa N. Revision of diagnostic guidelines for Kawasaki disease (the 5th revised edition). Pediatr Int. (2005) 47:232–4. 10.1111/j.1442-200x.2005.02033.x 15771703

[15] Kobayashi T Inoue Y Takeuchi K Okada Y Tamura K Tomomasa T. Prediction of intravenous immunoglobulinunresponsiveness in patients with Kawasaki disease. Circulation. (2006) 113:2606–12. 10.1161/CIRCULATIONAHA.105.592865 16735679

[16] JCS Joint Working Group. Guidelines for diagnosis and management of cardiovascular sequelae in Kawasaki disease (JCS 2013). Digest version. Circ J. (2014) 78:2521–62. 10.1253/circj.CJ-66-0096 25241888

[17] Ae R Makino N Kosami K Kuwabara M Matsubara Y Nakamura Y. Epidemiology, treatments, and cardiac complications in patients with Kawasaki Disease: The nationwide survey in Japan, 2017-2018. J Pediatr. (2020) 225:23–29. e2. 10.1016/j.jpeds.2020.05.034 32454114

[18] Surveillance data from Kitakyushu Institute of Health and Environmental Sciences. City of Kitakyushu (in Japanese). Available online at: https://www.city.kitakyushu.lg.jp/shisetsu/menu06_0140.html (accessed January 23, 2022).

[19] Abzug MJ Keyserling HL Lee ML Levin MJ Rotbart HA. Neonatal enterovirus infection: virology, serology, and effects of intravenous immune globulin. Clin Infect Dis. (1995) 20:1201–6. 10.1093/clinids/20.5.1201 7620000

[20] Zhang Y Moore DD Nix WA Oberste MS Weldon WC. Neutralization of Enterovirus D68 isolated from the 2014 US outbreak by commercial intravenous immune globulin products. J Clin Virol. (2015) 69:172–5. 10.1016/j.jcv.2015.06.086 26209401 PMC6512324

[21] Liu J Qi J. Prevalence and management of severe hand, foot, and mouth disease in Xiangyang, China, from 2008 to 2013. Front Pediatr. (2020) 8:323. 10.3389/fped.2020.00323 32754560 PMC7366859

[22] Galama JM Gielen M Weemaes CM. Enterovirus antibody titers after IVIG replacement in agammaglobulinemic children. Clin Microbiol Infect. (2000) 6:630–2. 10.1046/j.1469-0691.2000.00173.x 11168072

[23] Cellier C Foray S Hermine O. Regional enteritis associated with enterovirus in a patient with X-linked agammaglobulinemia. N Engl J Med. (2000) 342:1611–2. 10.1056/NEJM200005253422113 10841685

[24] Nakamura Y Yashiro M Uehara R Sadakane A Tsuboi S Aoyama Y. Epidemiologic features of Kawasaki disease in Japan: results of the 2009-2010 nationwide survey. J Epidemiol. (2012) 22:216–21. 10.2188/jea.JE20110126 22447211 PMC3798622

[25] Kim GB Park S Kwon BS Han JW Park YW Hong YM. Evaluation of the temporal association between Kawasaki Disease and viral infections in South Korea. Korean Circ J. (2014) 44:250–4. 10.4070/kcj.2014.44.4.250 25089137 PMC4117846

[26] Tsai CM Yu HR Tang KS Huang YH Kuo HC. C-reactive protein to albumin ratio for predicting coronary artery lesions and intravenous immunoglobulin resistance in Kawasaki disease. Front Pediatr. (2020) 8:607631. 10.3389/fped.2020.607631 33324592 PMC7723900

[27] Fortuna-Reyna B Bainto EV Ulloa-Gutierrez R Garrido-García LM Estripeaut D Del Águila O. Use of adjunctive therapy in acute Kawasaki disease in Latin America. Front Pediatr. (2020) 8:442. 10.3389/fped.2020.00442 33194876 PMC7646534

[28] Kent L McPherson M Higgins N. A positive association between cryptosporidiosis notifications and ambient temperature, Victoria, Australia, 2001-2009. J Water Health. (2015) 13:1039–47. 10.2166/wh.2015.130 26608765

[29] Jusot JF Neill DR Waters EM Bangert M Collins M Bricio Moreno L. Airborne dust and high temperatures are risk factors for invasive bacterial disease. J Allergy Clin Immunol. (2017) 139:977–86. 10.1016/j.jaci.2016.04.062 27523432 PMC5338876

[30] Claus H Vogel U Swiderek H Frosch M Schoen C. Microarray analyses of meningococcal genome composition and gene regulation: a review of the recent literature. FEMS Microbiol Rev. (2007) 31:43–51. 10.1111/j.1574-6976.2006.00047.x 17096662

